# HEART FAILURE, ARRHYTHMIAS AND DEMENTIA IN THE “OLDEST OLD” OF SOUTH CENTRAL UNITED STATES

**DOI:** 10.1093/geroni/igz038.3485

**Published:** 2019-11-08

**Authors:** Brandon Duke, Gohar Azhar, Amanda Pangle, Priya Mendiratta, Jeanne Wei

**Affiliations:** Donald W. Reynolds Institute on Aging, UAMS, Little Rock, Arkansas, United States

## Abstract

Heart failure and dementia are common age-related conditions. Heart failure and associated comorbidities of hypertension and arrhythmias may impact cognition. Retrospective data of 311 patients averaging 98 (±3.2) years who received treatment at the University of Arkansas for Medical Sciences were analyzed for diagnoses, prescribed medications and health conditions relevant to heart failure, hypertension, arrhythmias and dementia. 74% of the subjects were white, non-Hispanic, 24% were African American, and 2% were of unknown ethnicity. 83% were women and 17% were men. Only 251 (81%) of the reviewed charts had blood pressures recorded, of whom 43% (n=114) had systolic pressures >140mmHg. Furthermore, 50% (n=156) of patients had heart failure, and 29% (n=89) had dementia. Of those with dementia, 35% had an arrhythmia. For those without a diagnosis of dementia or any treatment for dementia, 25% had an arrhythmia. Heart failure and arrhythmias have not been well studied as an etiological factor for dementias. In our cohort, the presence of heart failure diagnoses was not different in those with dementia versus those without dementia. However, more patients with dementia had arrhythmias versus those without dementia, suggesting that arrhythmias may contribute to cognitive decline, even in the oldest old. Approximately 70% of the arrhythmias were atrial fibrillation. We did not have data on the management of these arrhythmias and whether anticoagulants were being used appropriately, especially for atrial fibrillation. Nevertheless this highlights the importance of close management of arrhythmias for maintaining cognitive health in older adults.

